# Incidence, Detection, and Management of Urological Injuries in Obstetric and Gynecologic Surgeries: A Five-Year Observational Study

**DOI:** 10.7759/cureus.96870

**Published:** 2025-11-14

**Authors:** Vinay S Kundargi, Kiran K Negi, Siddanagouda B Patil, Santosh Patil, Dhruva H M, Gulshan Kumar

**Affiliations:** 1 Urology, Shri B.M. Patil Medical College Hospital and Research Centre, Bijapur Lingayat District Educational Association (Deemed to be University), Vijayapura, IND

**Keywords:** bladder injury, gynecologic surgery, obstetric surgery, ureteric injury, urological injuries

## Abstract

Introduction: Urological injuries are rare but significant complications of obstetric and gynecologic procedures, associated with morbidity, prolonged hospital stay, and medicolegal implications. Bladder injuries are typically identified intraoperatively, while ureteric injuries are often delayed presentation. Early recognition and prompt management are critical to reduce complications. The study aimed to evaluate the incidence, type, timing of detection, management, and outcomes of urological injuries in patients undergoing obstetric and gynecologic surgeries.

Materials and methods: This retrospective and prospective study included 15,697 procedures (6150 gynecologic surgeries, 9547 lower segment cesarean sections) performed from January 2020 to December 2024 at a tertiary care center. Intraoperative and postoperative urological injuries were recorded and classified as early (≤7 days) or late (>7 days). Management strategies included primary repair, endoscopic procedures, stenting, or ureteric reconstruction. Descriptive statistics summarized patient demographics, injury rates, and outcomes. Comparisons were performed using chi-square and t-tests, with p <0.05 considered significant.

Results: Urological injuries occurred in 43 (0.27%) procedures; bladder injuries in 34 (0.21%), ureteric injuries in 7 (0.04%), and combined injuries in 2 (0.01%). Bladder injuries were slightly more frequent during obstetric procedures 22 (0.23%) compared to gynecologic procedures 12 (0.19%), whereas ureteric injuries occurred only in gynecologic surgeries 7 (0.11%). Most injuries 33 (76.7%) were detected intraoperatively. Intraoperative bladder injuries were repaired primarily with suprapubic cystostomy, and ureteric injuries underwent uretero-neocystostomy or ureteroureterostomy. Early recognition facilitated effective management and favorable outcomes, with mean hospital stay ranging from four to 12 days depending on injury type and timing.

Conclusion: Urological injuries are uncommon but clinically important. Early intraoperative detection and tailored surgical management ensure optimal outcomes. Preventive strategies, including meticulous surgical technique and careful intraoperative assessment, are essential to minimize morbidity.

## Introduction

Urological injuries represent one of the most significant complications encountered during obstetric and gynecologic surgeries, with reported incidences ranging from 0.3% to 1.5% [1]. These injuries are associated with high morbidity and potential medicolegal implications [2]. The incidence varies depending on the type of surgery and the surgeon's experience, yet their impact on patient quality of life and postoperative recovery is substantial [3]. Wong et al., in a 2018 systematic review of gynecologic laparoscopy for benign indications, reported a bladder injury incidence of 337 (0.24%) [4]. Such injuries are more frequently observed during complex pelvic surgeries, repeat procedures, or in patients with prior pelvic adhesions [5,6].

Urological complications can be broadly categorized into acute and chronic types. Bladder injuries are usually recognized intraoperatively and repaired immediately, often without the need for further intervention [2,7]. In contrast, ureteral injuries are frequently missed at the time of surgery and present later as chronic complications, such as urinoma, intra-abdominal abscess, ureteral strictures or fistulas, renal loss, sepsis, or even death [8]. Hysterectomy, particularly total abdominal and radical approaches, remains the most common procedure associated with iatrogenic urinary tract injuries in developed countries, with the bladder being the most frequently affected organ [9].

The increasing frequency of cesarean sections and complex pelvic surgeries in recent years has further contributed to the rising incidence of urological injuries [10]. Surgeon experience and surgical approach play critical roles in complication rates, with lower surgical volumes (<10 cases per year) being associated with a higher risk [11,12]. These factors highlight the importance of early recognition, timely intervention, and strategies to prevent injury during gynecologic and obstetric procedures. This study aims to determine the incidence and pattern of urological injuries associated with obstetric and gynecological procedures, evaluate the timing of their detection (early or delayed), and outline the management approaches and outcomes.

## Materials and methods

This retrospective and prospective observational study was conducted in the Department of Urology, Shri B.M. Patil Medical College, Hospital and Research Center, Vijayapura, over a five-year period from January 2020 to December 2024. We included all patients undergoing gynecologic and obstetric procedures during this period, comprising 6150 gynecologic surgeries and 9547 lower segment cesarean sections (LSCS). The study was approved by the Institutional Ethics Committee (IEC No: BLDE (DU)/IEC/1143/2024-25).

Cases were identified through operative theater logs, departmental databases, and medical record audits to ensure comprehensive case capture. Patients with pre-existing or previously treated urological injuries were excluded to avoid confounding of outcomes. We recorded all intraoperative and postoperative urological injuries. Intraoperative injuries included bladder or ureteric laceration, transection, rupture, or ligation, while postoperative complications included radiographically or clinically detected urinary leakage, ureteric obstruction, or fistula formation. Postoperative injuries were confirmed either clinically (based on urinary leakage or symptoms) or radiologically using ultrasonography, intravenous urography (IVU), or computed tomography (CT) urography, depending on the clinical scenario. Injuries detected within seven days of surgery were classified as early, and those detected thereafter as late. Intraoperative injuries were repaired immediately during the same surgery, while postoperative injuries were managed according to type and severity, including endoscopic procedures, stenting, ureteric reimplantation, or bladder repair with suprapubic catheterization.

Patient data, including age, parity, body mass index, previous abdominal or pelvic surgeries, type of procedure, and intraoperative findings, were collected from hospital records and operative notes. The incidence of urological injuries was analyzed according to surgical procedure, with particular focus on high-risk surgeries such as total abdominal hysterectomy (TAH) and laparoscopic hysterectomy. All patients were followed longitudinally with physical examination, laboratory investigations, and imaging studies, including ultrasound and intravenous urography, to evaluate the effectiveness of treatment and detect late complications.

Descriptive statistics were used to summarize continuous and categorical variables. Comparisons between gynecologic and obstetric procedures were performed using t-tests for continuous variables and chi-square tests for categorical variables. Data analysis was performed using IBM SPSS Statistics for Windows, Version 27.0 (IBM Corp., Armonk, NY, USA). P-values <0.05 were considered statistically significant.

## Results

The study included a total of 15,697 procedures, comprising 6150 (39.18%) gynecologic surgeries and 9547 (60.82%) lower segment cesarean sections (LSCS). The mean age of participants was 38.4 ± 12.6 years, with the gynecologic group being older (42.6 ± 10.3 years) than the obstetric group (34.2 ± 8.7 years, p < 0.001). The majority of participants were multiparous (gynecologic: 5338 (86.8%); obstetric: 8232 (86.2%); total: 13,570 (86.5%)), with no significant difference in parity between groups. Body mass index (BMI) categories, previous abdominal surgeries, socioeconomic status, and occupation were similar across the two groups, with no statistically significant differences (Table 1).

**Table 1:** Sociodemographic and clinical profile of the study population. Variables include age (in years), parity, body mass index (BMI, in kg/m²), history of previous abdominal surgeries, socioeconomic status, and occupation. Data are expressed as mean ± standard deviation (SD) for continuous variables and as frequency (percentage) for categorical variables. Statistical comparisons were made using Student's t-test for continuous variables and chi-square (χ²) test for categorical variables. BMI: body mass index; LSCS: lower segment cesarean section; SD: standard deviation.

Variable		Gynecologic procedures (n = 6150)	Obstetric procedures, LSCS (n = 9547)	Total (n = 15,697)	Statistical value	p-value
Age (years)	Mean ± SD	42.6 ± 10.3	34.2 ± 8.7	38.4 ± 12.6	t value = 12.48	<0.001
Parity	Nulliparous	812 (13.2%)	1315 (13.8%)	2127 (13.6%)	χ² = 1.0400 (df = 1)	0.3078
	Multiparous	5338 (86.8%)	8232 (86.2%)	13,570 (86.5%)		
BMI (kg/m²)	Normal (18.5–24.9)	2410 (39.2%)	3710 (38.9%)	6120 (39.0%)	χ² = 4.8214 (df = 2)	0.0897
	Overweight (25–29.9)	2710 (44.1%)	4110 (43.1%)	6820 (43.5%)		
	Obese (≥30)	1030 (16.8%)	1727 (18.1%)	2757 (17.6%)		
Previous abdominal surgery	Prior cesarean section	1480 (24.1%)	2280 (23.9%)	3760 (24.0%)	χ² = 0.0689 (df = 1)	0.7929
	Prior pelvic/gynecologic surgery	890 (14.5%)	1360 (14.3%)	2250 (14.3%)	χ² = 0.0689 (df = 1)	0.7929
Socioeconomic status	Upper/upper middle	1720 (28.0%)	2620 (27.4%)	4340 (27.7%)	χ² = 1.5178 (df = 2)	0.4681
	Lower middle	2580 (42.0%)	4100 (42.9%)	6680 (42.6%)		
	Upper lower/lower	1850 (30.1%)	2827 (29.6%)	4677 (29.8%)		
Occupation	Homemaker	3025 (49.2%)	4800 (50.3%)	7825 (49.9%)	χ² = 3.6975 (df = 2)	0.1574
	Employed (skilled/semi-skilled)	1960 (31.9%)	3050 (32.0%)	5010 (31.9%)		
	Professional	1165 (18.9%)	1697 (17.8%)	2862 (18.2%)		

Among gynecologic procedures, total abdominal hysterectomy (TAH) was the most commonly performed surgery (2460 (15.7%)), followed by vaginal hysterectomy (2033 (13.0%)) and laparotomy for ovarian cysts, ectopic pregnancy, or myomectomy (1323 (8.4%)). Non-descent vaginal hysterectomy (297 (1.9%)) and total laparoscopic hysterectomy/laparoscopic-assisted vaginal hysterectomy (37 (0.2%)) were less frequent. In the obstetric group, LSCS accounted for 9547 procedures (60.8% of total), making it the largest proportion of the surgical population (Table 2).

**Table 2:** Distribution of surgical procedures in the study population (n = 15,697). Gynecologic procedures include total abdominal hysterectomy (TAH), vaginal hysterectomy (VH), non-descent vaginal hysterectomy (NDVH), total laparoscopic hysterectomy (TLH), laparoscopic-assisted vaginal hysterectomy (LAVH), and laparotomy for ovarian cysts, ectopic pregnancy, or myomectomy. Obstetric procedures consist solely of lower segment cesarean sections (LSCS). TAH: total abdominal hysterectomy; VH: vaginal hysterectomy; NDVH: non-descent vaginal hysterectomy; TLH: total laparoscopic hysterectomy; LAVH: laparoscopic-assisted vaginal hysterectomy; LSCS: lower segment cesarean section.

Type of procedure	Surgical procedure	Frequency (n)	Percentage (%)
Gynecologic procedures	Total abdominal hysterectomy	2460	15.70%
	Vaginal hysterectomy	2033	13.00%
	Non-descent vaginal hysterectomy	297	1.90%
	Total laparoscopic hysterectomy/laparoscopic-assisted vaginal hysterectomy	37	0.20%
	Laparotomy (for ovarian cysts, ectopic pregnancy, myomectomy)	1323	8.40%
Obstetric procedures	Lower segment cesarean section	9547	60.80%
Total		15,697	100%

Urological injuries were observed in 43 of 15,697 procedures (0.27%). Bladder injuries occurred in 34 cases (0.21%), ureteric injuries in seven cases (0.04%), and combined bladder plus ureteric injuries in two cases (0.012%). Bladder injury incidence was comparable between gynecologic (12 (0.19%)) and obstetric procedures (22 (0.23%); p = 0.6422), whereas ureteric injuries were exclusively observed in gynecologic surgeries (7 (0.11%); p = 0.0009) (Table 3).

**Table 3:** Distribution of urological injuries. Injuries are classified as bladder, ureteric, or combined (both bladder and ureteric). The table includes total cases, percentage incidence, chi-square (χ²) values, and p-values indicating statistical significance between groups.

Type of injury	Gynecologic (n = 6150)	Obstetric (n = 9547)	Total (n = 15,697)	χ² value	p-value
Bladder injury	12 (0.19%)	22 (0.23%)	34 (0.21%)	0.2158 (df = 1)	0.6422
Ureteric injury	7 (0.11%)	0	7 (0.04%)	10.8713 (df = 1)	0.0009
Combined injury	2 (0.03%)	0	2 (0.012%)	3.1051 (df = 1)	0.0781
Total	21 (0.34%)	22 (0.23%)	43 (0.27%)	1.6877 (df = 1)	0.1939

The incidence of urological injuries varied according to surgical procedure. TAH had the highest number of bladder (9 (0.36%)) and ureteric injuries (5 (0.20%)), along with two combined injuries (0.08%). Laparoscopic-assisted vaginal hysterectomy (LAVH), although limited in number, had the highest proportion of injuries (two of 37 cases (5.4%)). Vaginal hysterectomy was not associated with any urological injuries. Non-descent vaginal hysterectomy and laparotomy had a minimal incidence. LSCS was associated with 22 bladder injuries (22/9547 (0.23%)) and no ureteric or combined injuries (Table 4).

**Table 4:** Incidence of urological injuries by type of surgery. Data are expressed as frequency (percentage) for categorical variables. TAH: total abdominal hysterectomy; VH: vaginal hysterectomy; NDVH: non-descent vaginal hysterectomy; TLH: total laparoscopic hysterectomy; LAVH: laparoscopic-assisted vaginal hysterectomy; LSCS: lower segment cesarean section.

Procedure	Cases (n)	Bladder injury n (%)	Ureteric injury n (%)	Combined injury n (%)	Total n (%)
Total abdominal hysterectomy	2460	9 (0.36%)	5 (0.20%)	2 (0.08%)	16 (0.65%)
Vaginal hysterectomy	2033	0	0	0	0
Non-descent vaginal hysterectomy	297	1 (0.33%)	0	0	1 (0.33%)
Total laparoscopic hysterectomy/laparoscopic-assisted vaginal hysterectomy	37	1 (2.70%)	1 (2.70%)	0	2 (5.40%)
Laparotomy (for ovarian cysts, ectopic pregnancy, myomectomy)	1323	1 (0.07%)	1 (0.07%)	0	2 (0.15%)
Lower segment cesarean section	9547	22 (0.23%)	0	0	22 (0.23%)
Total	15,697	34 (0.21%)	7 (0.04%)	2 (0.012%)	43 (0.27%)

Most urological injuries were detected intraoperatively (33 (76.7%)). Bladder injuries were identified intraoperatively in 27 of 34 cases (79.4%), while seven cases (20.6%) were detected within one to seven postoperative days. Ureteric injuries were detected intraoperatively in four of seven cases (57.1%) and postoperatively in three cases (42.9%). Both combined bladder and ureteric injuries (2 (100%)) were recognized intraoperatively (Table 5).

**Table 5:** Timing of detection of urological injuries. Data are expressed as frequency (percentage) for categorical variables. TAH: total abdominal hysterectomy; VH: vaginal hysterectomy; NDVH: non-descent vaginal hysterectomy; TLH: total laparoscopic hysterectomy; LAVH: laparoscopic-assisted vaginal hysterectomy; LSCS: lower segment cesarean section.

Time of detection	Bladder injury (n = 34)	Ureteric injury (n = 7)	Combined injury (n = 2)	Total (n = 43)
Intraoperative	27 (79.41%)	4 (57.14%)	2 (100%)	33 (76.7%)
Post-op (1–7 days)	7 (20.59%)	2 (28.57%)	0	9 (20.9%)
Post-op (>1 week)	0	1 (14.29%)	0	1 (2.3%)

Management of urological injuries was tailored according to the type and timing of detection. All intraoperative bladder injuries (27/27 (100%)) underwent primary repair with suprapubic cystostomy, whereas postoperative bladder injuries (7/34 (20.6%)) were managed with endoscopic fulguration plus catheter drainage (4/7 (57.1%)) or transvesical repair (3/7 (42.9%)). Intraoperative ureteric injuries (4/7 (57.1%)) were treated with uretero-neocystostomy (3/4 (75.0%)) or ureteroureterostomy (1/4 (25.0%)), and postoperative ureteric injuries (ureterovaginal fistula (UVF), 3/7 (42.9%)) were managed with cystoscopy and double J (DJ) stenting (2/3 (66.7%)) or uretero-neocystostomy (1/3 (33.3%)). Both combined injuries (2/2 (100%)) were repaired intraoperatively with bladder repair, suprapubic catheter, and ureteroureterostomy (Table 6).

**Table 6:** Management of urological injuries (n = 43). This table describes the management strategies for bladder, ureteric, and combined bladder plus ureteric injuries, based on the timing of detection (intraoperative or postoperative). It also includes the corresponding duration of hospital stay in days (mean and range). Management modalities include primary repair, suprapubic cystostomy, endoscopic fulguration with catheter drainage, transvesical repair, ureteroneocystostomy, ureteroureterostomy, cystoscopy with double J (DJ) stenting, and combined repairs where required. DJ: double J (ureteral stent); post-op: postoperative.

Type of injury	Timing of detection	Management	n (%)	Duration of hospital stay (days, mean (min–max))
Bladder injuries (n = 34)	Intraoperative (n = 27)	Primary repair with suprapubic cystostomy	27 (100%)	7 (5–10)
	Postoperative (n = 7)	Endoscopic fulguration + catheter drainage	4 (57.1%)	5 (4–6)
		Transvesical repair	3 (42.9%)	8 (7–10)
Ureteric injuries (n = 7)	Intraoperative (n = 4)	Ureteroneocystostomy	3 (75.0%)	10 (8–12)
		Ureteroureterostomy	1 (25.0%)	9 (9–9)
	Postoperative (n = 3)	Cystoscopy + DJ stenting	2 (66.67%)	4 (3–5)
		Ureteroneocystostomy	1 (33.33%)	11 (11–11)
Combined bladder + ureteric injuries (n = 2)	Intraoperative (n = 2)	Bladder repair + suprapubic catheter + ureteroureterostomy	2 (100%)	12 (11–13)

## Discussion

The anatomical proximity of the lower urinary tract to the female reproductive system predisposes it to iatrogenic injuries during gynecologic and obstetric surgeries [3]. In the present study, the overall incidence of urological injuries was 43/15,697 (0.27%), with bladder injuries accounting for 34/15,697 (0.21%), ureteric injuries for 7/15,697 (0.04%), and combined bladder and ureteric injuries for 2/15,697 (0.01%). These findings are lower than the incidence reported in previous literature, which ranges from 0.5% to 1.5% for major urological injuries during pelvic surgeries. Samal et al. reported that among 3166 gynecologic procedures, there were 16 (0.5%) bladder injuries and 1 (0.03%) ureteric injury, while among 5429 obstetric procedures, bladder injuries occurred in 11 (0.2%) cases and ureteric injuries in 1 (0.01%) cases, which is broadly consistent with our observations [13]. The relatively low incidence in our study may reflect early recognition, meticulous surgical technique, and inclusion of both elective and emergency cases in a high-volume tertiary care setting.

Bladder injuries were more common during obstetric procedures (22 (0.23%)) than gynecologic surgeries (12 (0.19%)), consistent with prior reports demonstrating higher bladder injury rates during cesarean sections compared to hysterectomies for benign indications [14,15]. Most bladder injuries were detected intraoperatively (27 (79.4%)), highlighting the importance of vigilant surgical technique and early recognition. Intraoperative detection allows immediate repair, reducing morbidity, shortening hospital stay, and minimizing medicolegal consequences [16,17].

Ureteric injuries were observed exclusively during gynecologic procedures, predominantly during total abdominal hysterectomy, with an incidence of 5 (0.20%). Laparoscopic-assisted procedures, although limited in number, showed a disproportionately high injury rate (2 (5.4%)), reflecting the technical complexity of minimally invasive surgery and the learning curve associated with advanced laparoscopy. These findings align with published data, indicating higher ureteric injury risk in complex gynecologic surgeries such as abdominal hysterectomy, radical pelvic surgeries, and cases with dense adhesions or endometriosis [18-20]. The intraoperative identification rate for ureteric injuries in our cohort was 4 (57.1%), higher than previously reported rates of around 30%, highlighting the value of meticulous surgical technique and intraoperative vigilance [21].

Management strategies in our study were tailored to the type and timing of injury. All intraoperatively detected bladder injuries (27 (100%)) underwent primary repair with suprapubic cystostomy, whereas postoperative bladder injuries (7 (20.6%)) were managed with endoscopic fulguration and catheter drainage (4 (57.1%)) or transvesical repair (3 (42.9%)). Intraoperative ureteric injuries (4 (57.1%)) were treated with uretero-neocystostomy (3 (75.0%)) or ureteroureterostomy (1 (25.0%)), and postoperative ureteric injuries (3 (42.9%)) were managed with cystoscopy and DJ stenting (2 (66.7%)) or delayed uretero-neocystostomy (1 (33.3%)). Both combined injuries (2 (100%)) were recognized and repaired intraoperatively. These management approaches are consistent with current recommendations that emphasize early recognition, prompt repair, and the involvement of urology specialists when necessary [16,17,22]. Our findings also reinforce that bladder injuries are approximately five times more common than ureteric injuries, but early intraoperative diagnosis facilitates effective management and favorable outcomes. Meticulous surgical technique, intraoperative vigilance, and patient counseling remain key to preventing urological injuries, especially in high-risk gynecologic and obstetric procedures.

The major strengths of this study include its large sample size, the combined retrospective-prospective design, and comprehensive data capture covering both gynecologic and obstetric procedures. The inclusion of detailed intraoperative and postoperative injury classifications, timing of detection, and management strategies enhances its real-world applicability and clinical relevance. Furthermore, the use of standardized documentation and imaging confirmation improves the reliability and reproducibility of results.

This study has several limitations. Being a single-center observational study, its findings may not be generalizable to all settings. The number of laparoscopic procedures was relatively small (37 (0.2%)), limiting conclusions regarding minimally invasive surgery-related injuries. Additionally, long-term follow-up was not uniform for all patients, potentially underestimating late complications such as ureteral strictures or vesicovaginal fistula formation. There is also potential for reporting bias inherent to retrospective data collection, as minor injuries or cases managed conservatively may have been underreported. The study did not perform multivariate analysis to identify independent predictors of urological injury, limiting the assessment of confounding variables. Moreover, long-term functional outcomes, such as renal function or quality of life, were not evaluated, which could have added further clinical depth.

## Conclusions

Urological injuries during gynecologic and obstetric surgeries are uncommon but clinically significant, with bladder injuries being more frequent than ureteric injuries in our study. Total abdominal hysterectomy and emergency lower segment cesarean sections were associated with the highest incidence, while most injuries were identified intraoperatively and managed effectively. Early recognition and appropriate surgical intervention resulted in favorable outcomes, highlighting the importance of surgical expertise, careful dissection, and intraoperative vigilance. Implementation of preventive strategies, including meticulous technique, cystoscopic evaluation in high-risk cases, and timely postoperative assessment, is essential to minimize morbidity and improve patient safety. The findings of this study demonstrate associations between surgical procedures and the occurrence of urological injuries rather than direct causal relationships. Future multicenter and large-scale prospective studies are warranted to validate these observations, identify predictive factors, and further refine preventive and management strategies.
